# Enhancing quality of life among head and neck cancer survivors with nurse-led care during radiotherapy

**DOI:** 10.6026/973206300212712

**Published:** 2025-08-31

**Authors:** Padmavathy Murugan, Vijayasree T.N., Shankar Shanmugam Rajendran, Duraikannu Anandhi, Vasanth Pandian Thommai Antony Savari Muthu, Pradeepa Govindharaj, Athya Mohammed Sulthan

**Affiliations:** 1 Department of Medical Surgical Nursing, College of Nursing, Madras Medical College, Tamil Nadu Dr .MGR Medical University, Chennai, Tamil Nadu, India; 2 Department of Radiation Oncology, Madras Medical College & RGGGH, Chennai, Tamil Nadu, India; 3 Department of Child Health Nursing, College of Nursing, Madras Medical College, Chennai, Tamil Nadu, India; 4 Department of Medical Surgical Nursing, College of Nursing, Madras Medical College, Chennai, Tamil Nadu, India

**Keywords:** Nurse-led survivorship care, head and neck cancer, pain, fatigue, caregiver experience, radiation therapy

## Abstract

The ongoing physical, emotional, and social challenges faced by head and neck cancer (HNC) survivors and their caregivers, particularly after completing treatment is of interest. It should be noted that traditional medical care often overlooks this issue. This study evaluated the effectiveness of nurse-led survivorship care for patients with head and neck cancer (HNC) in managing pain, fatigue and supporting caregivers. A 21-day intervention program, comprising exercises, therapy and educational sessions, significantly reduce pain and fatigue in the experimental group (p < 0.05). Caregiver interviews revealed emotional stress, lack of preparedness and a need for structured support. The intervention helped caregivers cope better by providing guidance and support. Incorporating nurse-led survivorship care into routine practice can significantly improve patient recovery and overall survivorship outcomes.

## Background:

Head and neck cancers affect vital functions like speaking, swallowing and breathing, making them particularly challenging [1]. In India, their rising incidence is linked to tobacco use, vaping, alcohol and poor lifestyle habits [2]. Even after completing treatment, many survivors face ongoing issues such as pain, fatigue, dry mouth and speech difficulties, which impact their daily life and emotional well-being [3]. Recovery doesn't end with radiation therapy. Patients and caregivers continue to face physical, emotional and financial stress. While traditional care often focuses on medical treatment, it usually overlooks the emotional and social needs of both patients and caregivers [4]. Nurse-led survivorship care addresses this gap by offering consistent support, symptom relief, education and emotional counselling [5]. Through simple activities such as seated exercises, reminiscence therapy and games, nurses can help reduce fatigue and boost patients' moods. Caregivers also benefit from guidance and emotional support, which allows them to cope better [6]. Therefore, it is of interest to describe the use of structured, nurse-led approach to alleviate pain and fatigue and provide support to caregivers, and enhance the overall quality of life for head and neck cancer survivors.

## Methods and Materials:

An explanatory sequential mixed-method design was used to assess the effectiveness of nurse-led survivorship care on pain, fatigue and caregiver experience among head and neck cancer patients undergoing radiation therapy. The study was conducted in the Radiation Oncology Department of Rajiv Gandhi Government General Hospital, Chennai, after receiving ethical clearance from the Institutional Ethics Committee of Madras Medical College and approval from the Dean. In the qualitative phase, five caregivers were selected using convenience sampling and interviewed using a semi-structured guide to explore their caregiving experiences. Data were recorded, transcribed and analysed thematically. The insights gained helped shape the nurse-led intervention. In the quantitative phase, 60 head and neck cancer patients were chosen by purposive sampling and evenly divided into experimental and control groups. Participants were adults receiving radiation therapy and willing to participate. Critically ill patients and those unwilling to give consent were excluded. The experimental group received a 21-day structured intervention including seated exercises, reminiscence therapy, board games and educational sessions. The control group received routine care. Pain and fatigue were measured using the Numerical Pain Rating Scale and the Fatigue Scale. Data were analysed using SPSS software. Descriptive and inferential statistics were applied and a p-value ≤ 0.05 was considered statistically significant.

## Results:

Among the participants, 50% were male, with the majority aged between 51-60 years (36.67%). Most had primary education (50%) and were undergoing both chemotherapy and radiation. Groups were matched at baseline, with no significant differences in demographic variables. Qualitative analysis revealed six primary themes: (1) Expectations vs. Reality of Care, (2) Pain and Symptom Management, (3) Barriers in Care Delivery, (4) Communication Gaps, (5) Caregiver Stress and Coping and (6) Learning and Adaptation. These highlighted emotional strain, limited guidance and the positive impact of structured nurse-led care. At pre-test, pain scores were high and similar in both groups. Post-intervention, 70% of the experimental group reported mild pain compared to 0% in the control group. The experimental group showed a 43.60% reduction in pain, which statistically significant (P < 0.001) was shown in Table 1 (see PDF). Pre-test scores were comparable. After intervention, 73.33% of the experimental group had low fatigue levels, while 43.33% in the control group reported high fatigue. A 34.60% fatigue reduction was observed in the experimental group (P < 0.001) as shown in Table 2 (see PDF). There was a moderate positive correlation between pain and fatigue in the experimental group (r = 0.41, P = 0.001), indicating that a reduction in one was associated with a reduction in the other. Better outcomes were seen in participants aged 51-60, those with higher education and those in early cancer stages. However, these trends were not statistically significant in the control group as shown in Table 3 (see PDF).

## Discussion:

This study showed that nurse-led survivorship care helped reduce pain and fatigue in head and neck cancer patients receiving radiation therapy. These results match with previous studies, like Lee *et al.* (2023) [7], who found that nurse-led support improves both physical symptoms and emotional well-being. The positive link between pain and fatigue observed here is also supported by Liu *et al.* (2022) [8], who highlighted the close connection between these symptoms during cancer recovery. The caregiver interviews revealed emotional challenges, lack of guidance and stress, which agree with findings from Li *et al.* (2023) [9] who stressed the need for more emotional and practical support for caregivers. Similarly, Martinez *et al.* (2015) [10] demonstrated that nurse-led phone support can reduce symptoms and decrease hospital visits. Additionally, Rajendran *et al.* (2021) [11] explored the well-being of breast cancer survivors and found that emotional challenges such as fear, loneliness and low self-esteem significantly affected recovery, further supporting the need for psychosocial care. Altogether, this study supports including nurse-led care in routine cancer management. It not only controls symptoms but also helps caregivers cope better. Future research should focus on long-term outcomes and explore ways to apply this model more broadly.

## Conclusion:

The study demonstrated that nurse-led survivorship care effectively reduces pain and fatigue and enhances patient well-being. Additionally, it supports caregivers, highlighting its value in promoting holistic recovery for head and neck cancer survivors.
